# Editorial: Neuroplasticity in cognitive and psychological mechanisms of anxiety

**DOI:** 10.3389/fnmol.2023.1241279

**Published:** 2023-07-03

**Authors:** Massimo Grilli, Ismail Zaed

**Affiliations:** ^1^Department of Pharmacy, University of Genoa, Genoa, Italy; ^2^Department of Neurosurgery, Neurocenter of South Switzerland, EOC, Lugano, Switzerland

**Keywords:** anxiety, brain derived neurotrophic factor (BDNF), exercise, autism (ASC), neuropathic pain (NP), amygdala, microbiota, methamphetamine

The impact of anxiety in contemporary society is increasing. Several new impulses emerged from the modern types of anxiogenic stimuli: lockdown-anxiety, eco-anxiety and climate-anxiety (Clayton, 2020; Panu, 2020; Coffey et al., 2021). Anxiety disorders affect both male and female patients, but women are twice the risk for developing anxiety and depression than men (Asher et al., 2017). The complexity of the cellular and molecular elements involved in aggravating and maintaining this state is partly explained by the fact that it can represent a contemporary symptom, comorbidity, or pathological condition. For example, emerging evidence shows a link between unexpected diseases, such as neuropathic pain, vascular dementia and autism, and anxiety (Ballard et al., 2000; Seignourel et al., 2008; White et al., 2009; Gormsen et al., 2010). From a mechanistic point of view, a link between microbiota dysbiosis and anxiety seems to be confirmed (Clapp et al., 2017; Jiang et al., 2018). Several lines of work converge on a conserved set of brain regions required for the execution of adaptive defensive responses in both human and animal models. This circuitry includes the amygdala and other subcortical structures, which are necessary for the identification and coordination of behavioral and physiological responses to threats (Rauch et al., 2003; Walf and Frye, 2006). Nevertheless, international research provided data supporting escape strategies and pharmacological and non-pharmacological approaches to limit anxiety disorders. Digital mental health interventions and treadmill exercise have received interest from the public as well as the scientific community (Ströhle, 2009; Jayakody et al., 2014; Firth et al., 2018).

In this Research Topic, novel evidence is being discussed to supply a thorough overview in the field of Molecular Neuroscience and Anxiety and on its future challenges (Figure 1).

**Figure 1:**
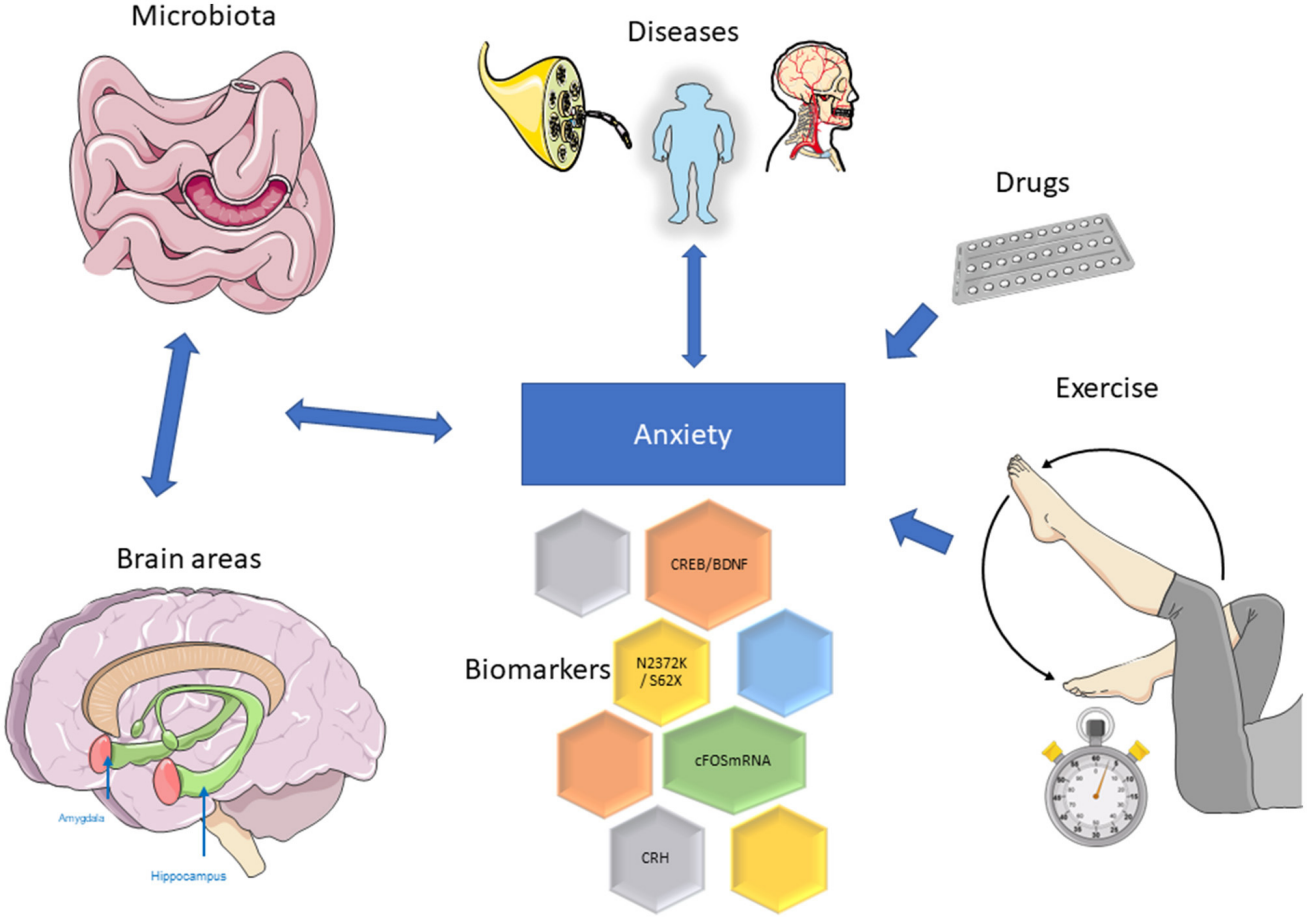
Representative image of data presented; for details, see main text. The figure was partly generated using Servier Medical Art, provided by Servier, licensed under a Creative Commons Attribution 3.0 unported license (https://smart.servier.com/, accessed June 14, 2023).

Herrera-Rivero et al. investigated the effects of genetic background and environmental enrichment on transcriptional profiles of the mouse amygdala using an established cognitive bias test. They observed wide-ranging molecular effects of genetic background in both living environments (normal and enriched). C57BL/6J animals showed more transcriptional changes in response to enriched environments than B6D2F1N mice. The authors found more dysregulated genes in the posterior than in the anterior part of the amygdala. Interestingly, though, strain-specific differences between both portions of the amygdala focused on stress and immune pathways, suggesting that these mouse strains may respond differently to environmental stimuli. Their results suggest the involvement of lipid metabolism in optimistic cognitive bias and, in general, propose a crucial role for immunity in the control of the amygdala-related emotional processing.

Wen et al. analyzed the link between neuropathic pain and anxiety. They show that late-stage neuropathic pain is associated with anxiety and depression. Silencing of the anterior cingulate cortex resulted in a significant alleviation of pain sensitivity, anxiety, and depression in rats with sparing nerve injury. Mechanistically, the CREB/BDNF signaling pathway was activated and central and peripheral inhibition of CREB reversed pain sensitivity and anxiety disorders caused by peripheral nerve injury. Therefore, the authors hypothesize that cingulate CREB/BDNF regulation could be a safe therapeutic method for the treatment of neuropathic pain and associated stress.

Re et al. investigated the effects of exercise as a therapeutic program to reduce anxiety-like symptoms in acute withdrawal methamphetamine mice and users. They found severe peripheral immune dysfunction in methamphetamine users during acute withdrawal. This significant inflammatory response may contribute in part to anxiety symptoms. As shown in a constructed mouse model, the mouse striatum and hippocampus showed microglial activation and proinflammatory cytokines release during acute withdrawal of methamphetamine. Treadmill exercise attenuated the anxiety-like symptoms induced by methamphetamine acute withdrawal. Accordingly, treadmill exercise counteracted methamphetamine-induced microglial activation and increased the release of proinflammatory cytokines. This report provides new data showing the immunomodulatory modulation of specific targets during exercise in male patients affected by acute anxiety induced by withdrawal from methamphetamines.

Lee et al. analyzed autism-like behavior in Chd8 mutant mice (knock-in). Juvenile Chd8 mice exhibited sex and age-dependent behaviors. Taking into account the anxiety-related symptoms, Chd8+/S62X juveniles show increased mother seeking, which is followed by increased anxiety-like behavior in adults (decreased open-field center time [females] and increased closed-arm time [males and females]. Moreover, this study shows that two different mutations (N2372K vs. S62X) can change the extent and time course of sexual dimorphisms in autistic-like phenotypes.

Shu et al. performed a bioinformatic analysis of the frontal and temporal cortex in vascular dementia. They found overlapping differentially expressed genes (DEGs) between the frontal cortex and the temporal cortex in vascular dementia patients. Moreover, the authors recognized 10 hub genes (GNG13, CD163, C1QA, TLR2, SST, C1QB, ITGB2, CCR5, CRH, and TAC1), four key regulatory transcription factors (FOXC1, CREB1, GATA2, and HINFP), and four microRNAs (miR-27a-3p, miR-146a-5p, miR-335-5p, and miR-129-2-3p). CRH encodes a member of the corticotropin-releasing factor family that acts as an important regulator of homeostasis, mediating autonomic, behavioral, and neuroendocrine responses to stress. These results may help to understand the mechanisms of vascular dementia, and the early symptoms and provide potential targets and drugs for therapeutic interventions.

Pate et al. studied the relationships between brain plasticity and immune gene expression, peripheral immunity, and brain and liver metabolism in germ-free and specific pathogen-free mice. They investigated intermediary factors involved in the gut microbiota to brain communication, with implications for the role in anxiety. The main results of this work showed that brain acetate was significantly reduced in germ-free mice, while glutamate, glutamine and N-acetylaspartate metabolites were increased. Interestingly, cFOSmRNA expression, which was significantly reduced in the prefrontal cortex of germ-free mice, was correlated with glutamate and glutamine level. The study supplies insight into possible mechanisms by which the microbiota may regulate neurotransmission through modulation of the host's brain and liver metabolome, which may have implications for stress-related psychiatric disorders such as anxiety.

## Author contributions

MG and IZ contributed to conception and design of the study. MG wrote the first draft of the manuscript. All authors contributed to manuscript, read, and approved the submitted version.
